# Reference Gene Validation for Quantitative PCR Analysis in 2D and 3D AML12 Hepatocyte Models

**DOI:** 10.3390/biomedicines14010150

**Published:** 2026-01-11

**Authors:** Zhenya Ivanova, Valeria Petrova, Betina Todorova, Toncho Penev, Natalia Grigorova

**Affiliations:** 1 Department of Pharmacology, Animal Physiology, Biochemistry and Chemistry, Faculty of Veterinary Medicine, Trakia University, 6000 Stara Zagora, Bulgaria; zhenya.ivanova.12@trakia-uni.bg (Z.I.); valeriya.petrova@trakia-uni.bg (V.P.); betina.todorova@trakia-uni.bg (B.T.); 2 Department of Ecology and Animal Hygiene, Faculty of Agriculture, Trakia University, 6000 Stara Zagora, Bulgaria; toncho.penev@trakia-uni.bg

**Keywords:** reference genes, RT-qPCR normalization, 2D/3D cell culture, AML12 cells, in vitro hepatocyte model

## Abstract

**Background/Objectives**: Advanced 3D cell culture techniques enhance the physiological relevance of in vitro models, while supporting the 3Rs principles (Reduction, Refinement, and Replacement) of animal experimentation. In this context, 3D collagen-based systems mimic key extracellular matrix properties, enabling more accurate cellular organization and phenotype. However, changes in culture dimensionality can affect RT-qPCR reference gene stability, underscoring the need for careful validation when combining 2D and 3D systems. **Methods**: AML12 cells were cultured for 7 days under different 2D and collagen-based 3D conditions. The expression stability of nine candidate housekeeping genes was systematically evaluated using established algorithms (BestKeeper, NormFinder, geNorm, RefFinder, and ΔCt method), followed by inter-group statistical and correlation analyses of raw Ct values. Albumin gene expression was used as a target gene. **Results**: Although all candidate genes initially met acceptable variability thresholds, a stepwise, exclusion-based analysis revealed distinct performance differences. Hprt, Ppia, and Actb emerged as the most stable, showing no intra-group variability or interaction with Albumin expression. Nevertheless, Ywhaz and Rplp0, despite their high stability, were compromised by significant correlation with Albumin. Furthermore, Ywhaz showed significant downregulation under 3D culture conditions. B2M, Gapdh, 18S, and Hmbs exhibited increased variability, likely reflecting metabolic and microenvironmental heterogeneity associated with prolonged 2D cultivation of AML12 cells. **Conclusions**: Overall, this study highlights the importance of context-dependent, exclusion-based reference gene validation when comparing 2D and 3D models, and demonstrates a new approach for reliable gene expression normalization in complex in vitro culture systems.

## 1. Introduction

The liver is a vital metabolic organ in the human body, responsible for over 500 physiological and biochemical functions. It plays a key role in the synthesis of plasma proteins, regulation of glucose homeostasis, detoxification of foreign substances, and metabolization of lipids and pharmaceuticals [1,2,3,4,5,6,7]. Impairments in hepatic function exert a profound impact on the body’s homeostatic processes, highlighting the need for reliable in vitro models for the study of hepatic physiology and pathophysiology.

Primary hepatocytes are considered the most physiologically relevant in vitro model, as they closely resemble native liver tissue. However, their broader application is limited by restricted availability. The rapid loss of liver-specific functions following isolation renders them unsuitable for long-term experimental studies. Most in vitro studies rely on immortalized hepatic cell line culture systems, which remain essential tools for investigating liver function and disease [8,9]. The AML12 cell line is a stable, non-tumorigenic mouse hepatocyte model widely used for in vitro studies of liver disease, metabolism, and toxicology [10,11]. Unlike tumor-derived cell lines, such as HepG2 and Huh-7, AML12 preserves essential hepatocyte functions, including albumin production, urea synthesis, and cytochrome P450 enzyme activity [12,13,14].

Traditionally, AML12 cells are cultured under two-dimensional (2D) conditions, where they grow as a monoculture monolayer [15,16]. Although these systems are convenient, cost-effective, easy to maintain, and support high cell viability, 2D culture is limited by its inability to reproduce the complex intercellular interactions and signaling pathways characteristic of in vivo liver tissue [17,18,19]. Moreover, upon reaching full confluence, hepatocytes cultured in 2D systems undergo alterations in gene expression, morphology, and functional activity [16,19,20]. To address these limitations, three-dimensional (3D) cell culture and co-culture models, such as static hanging-drop suspension, liquid overlay techniques, spinner cultures, hydrogel-embedded cells, nanofibrous scaffolds, decellularized biological matrices, and 3D-printed constructs are employed to create microenvironments that resemble physiological tissue more closely [21,22,23,24,25]. The implementation of these innovative cell-culture techniques not only enhances the physiological relevance of in vitro models, but also bridges the gap between conventional in vitro systems and native tissue architecture, offering new opportunities for studying liver physiology, regeneration, and toxicology in accordance with the ethical principles of the 3Rs (Reduction, Refinement, Replacement) [26]. The 3Rs framework, introduced by Russell and Burch in 1959, laid the foundation for ethical animal research, while more recent concepts, such as the “Three Cs”-cell culture, computer simulation, and phase 0 clinical trials-offer complementary non-animal alternatives to improve the ethical and translational value of preclinical research [27,28]. Although some animal studies remain indispensable, ongoing advances in biomaterials, computational modeling, and artificial intelligence are rapidly accelerating the development of reliable alternatives.

Type I collagen is widely preferred as a 3D cell culture matrix and is often used as the sole or primary structural component of natural hydrogels due to its excellent biocompatibility, pronounced hydrophilicity, and intrinsically low antigenicity. As a key component of the natural extracellular microenvironment, it provides not only mechanical integrity, but also essential biochemical cues that support hepatocyte polarization, enhance albumin production, and promote regenerative capacity, thereby creating a microenvironment conducive to maintaining liver-specific functions in vitro [29,30]. In in vitro studies, collagen type I is often used in 2D cultures, where it primarily serves as a surface coating that facilitates adhesion, but under these culture conditions, it offers limited spatial signaling. In contrast, 3D collagen structures facilitate multidirectional cell–matrix interactions, thereby better preserving hepatocyte morphology, functional activity, and phenotypic stability. In these 3D systems, cells retain their regenerative potential longer and maintain stable expression of hepatocytic progenitor markers [31].

To demonstrate and optimize the ability of 3D in vitro systems to better mimic in vivo conditions, conventional 2D cultures are commonly used as reference controls, making comparative 2D–3D analyses central to validating functional and physiological improvements. Such experiments reveal pronounced differences in cellular organization, microenvironmental cues, and functional state between 2D and 3D cultures, which inevitably influence gene expression profiles. Consequently, a key challenge in the comparative analysis of gene expression across different culture dimensionalities is the accurate normalization of real-time quantitative PCR (RT-qPCR) data. The reliability of these results critically depends on the selection of appropriate reference (housekeeping) genes whose expression remains stable, irrespective of culture conditions [32,33,34]. Widely utilized HKGs involve: 18S rRNA (18S), ribosomal protein, large, P0 (Rplp0), β-actin (Actb), glyceraldehyde-3-phosphate dehydrogenase (Gapdh), hypoxanthine guanine phosphoribosyl transferase (Hprt), peptidylprolyl isomerase A (Ppia), β-2 microglobulin (B2M), TATA-box binding protein (Tbp). 18S encodes a core structural component of the small ribosomal subunit and is indispensable for protein synthesis [35]. Rplp0 is part of the 60S ribosomal subunit and contributes to translational efficiency [36]. Actb (β-actin) encodes a major cytoskeletal protein crucial for maintaining cell shape and structural integrity, while Gapdh functions as a key glycolytic enzyme involved in cellular energy metabolism [37,38]. The Ppia gene encodes cyclophilin A, which facilitates protein folding and regulates multiple signaling pathways [39]. B2M encodes β-2-microglobulin, an invariant component of MHC class I molecules broadly expressed across nucleated cells [40]. Hprt participates in the purine salvage pathway, supporting nucleotide recycling and genomic stability [41], whereas Tbp is a core transcription factor that binds to TATA promoter elements and is required for initiation of transcription by RNA polymerase II [42]. Collectively, these genes are frequently chosen for reference gene normalization due to their basal roles in ribosome function, cytoskeletal maintenance, metabolism, protein homeostasis, nucleotide salvage, and basal transcription.

Despite their common use as internal controls in 2D hepatic models, Gapdh and Actb have repeatedly been shown to exhibit condition-dependent variability, particularly under hypoxia, oxidative stress, or during the transition from 2D to 3D culture systems [43,44]. In hepatocyte-derived models, such as HepaRG, the expression of these genes varies markedly in response to experimental treatments. This variability can be attributed, in part, to the direct involvement of Gapdh in central metabolic pathways that are highly sensitive to changes in oxygen [45,46] availability and redox balance. Similarly, Actb encodes a major cytoskeletal protein whose expression dynamically responds to alterations in cell morphology, mechanical tension, and extracellular matrix organization factors that differ substantially between 2D monolayers and 3D collagen-based environments [45,46]. Such context-dependent regulation challenges the reliability of universal reference standards and highlights the need for empirical validation of HKGs within specific experimental setups. To date, systematic evaluations of optimal HKGs for AML12 cells, particularly within 3D culture systems are largely underperformed. In most studies involving AML12 cells, reference genes are selected empirically, relying on those most commonly reported in the literature, rather than being subjected to context-specific validation [10,47,48,49].

Accordingly, the aim of the present study is to comprehensively evaluate and validate a panel of commonly used RT-qPCR reference genes to identify those that provide the highest accuracy and reproducibility for gene-expression analysis in AML12 cells, with particular emphasis on how their stability is influenced by the mode of culture, 2D versus 3D.

## 2. Materials and Methods

### 2.1. Cell Line Propagation and Culture Conditions

The hepatic parenchymal cell line AML12 (300643, Cytion, Heidelberg, Germany) was maintained under standard culture conditions in a 1:1 mixture of Dulbecco’s modified Eagle’s medium (DMEM) and Ham’s F12 medium (Cytion, Heidelberg, Germany) supplemented with 10% fetal bovine serum (FBS), 1% insulin–transferrin–selenium (ITS, I3146, 100×), 1% antibiotic–antimycotic solution (penicillin, streptomycin, amphotericin B), and dexamethasone (D4902, 40 ng/mL) (all from Sigma-Aldrich Chemie GmbH, Merck KGaA, Darmstadt, Germany) to support hepatocyte-like characteristics. Cells were incubated at 37 °C in a humidified atmosphere of 5% CO_2_. Cultures were expanded in T75 tissue-culture flasks and passaged upon reaching approximately 80% confluence.

### 2.2. Two-Dimensional and Three-Dimensional Culture Systems

The mouse hepatocytes were then seeded in 24-well plates over a period of 7 days. AML12 cells were passaged twice after delivery and experiments were conducted at passage 2. The study was designed to compare several 2D culture protocols with a 3D approach. For all 2D protocols, cells were seeded at a density of 2 × 10^4^ cells per well. In the 2D-TC protocol, cells were cultured on standard tissue culture-treated plastic surfaces (Corning Inc., Corning, NY, USA). In the 2D-GelyP group, wells were coated with 0.1% gelatin-peptone (Gelysate Peptone, Gibco, Thermo Fisher Scientific, Waltham, MA, USA) in PBS and allowed to air-dry before seeding. For the 2D-Coll protocol, wells were coated with a thin layer of type I collagen (rat tail, Corning Inc., Corning, NY, USA) at 5 µg/mL, prepared in 0.02 N acetic acid, as per the Corning protocol. For 2D-GelyP and 2D-Coll protocols, non-treated plates were employed (Falcon, Corning Inc., Corning, NY, USA).

Among the 3D approaches, the sandwich method was selected, as the cells were seeded between two layers of type I collagen, with the basal layer at 2 mg/mL and the top layer at 1.5 mg/mL, according to the Corning protocol at a cell density of 3 × 10^4^/well. The experimental design allowed us to directly compare the morphology of hepatocytes in 2D and 3D cultures, using images taken with a Leica inverted microscope equipped with a 5-megapixel DMi1 camera (Leica Microsystems, Wetzlar, Germany).

### 2.3. RNA Extraction and cDNA Synthesis

At day 7, total RNA was extracted using the RNeasy Plus Mini Kit (cat. no. 74134 and 74136, Qiagen GmbH, Hilden, Germany) following the manufacturer’s protocol. RNA concentration and purity were evaluated spectrophotometrically by measuring absorbance at 260 and 280 nm with a Synergy™ LX Multi-Mode Microplate Reader equipped with a Take3 Microvolume Plate (BioTek Instruments, Inc., Winooski, VT, USA). All RNA samples were subsequently normalized to an equivalent concentration prior to reverse transcription. Complementary DNA (cDNA) was synthesized using the RevertAid First Strand cDNA Synthesis Kit (K1612, Thermo Scientific, Waltham, MA, USA), and the resulting cDNA samples were stored at −20 °C until further analysis.

### 2.4. Primer Design and Validation

Primer pairs for housekeeping and target genes were designed and evaluated using a combination of online bioinformatic tools. Initial primer sequences were generated through NCBI resources and Primer3, following the design guidelines recommended for SYBR Green–based qPCR assays. The 18S primer sequence was first reported by Arnhold et al. [50]. Primer specificity was confirmed using Primer-BLAST (NCBI) (https://www.ncbi.nlm.nih.gov/tools/primer-blast/, accessed on 21 October 2024) and Primer3Plus (version 3.2.5) (https://www.bioinformatics.nl/cgi-bin/primer3plus/primer3plus.cgi, accessed on 21 October 2024), while predicted amplicon properties-including product size, melting temperature, and theoretical melt-curve behavior-were assessed using Primer3 (https://primer3.ut.ee/, accessed on 21 October 2024) and uMelt Quartz (version 3.6.2) (https://www.dna-utah.org/umelt/quartz/um.php, accessed on 22 October 2024). The final primer sequences adopted in this study are provided in Table 1.

### 2.5. Quantitative Real-Time PCR (qPCR)

Quantitative real-time PCR was performed using a SYBR Green–based two-step amplification protocol with the KAPA SYBR^®^ Fast qPCR Master Mix (Kapa Biosystems, Merck KGaA, Darmstadt, Germany) on a PikoReal Real-Time PCR System (Thermo Scientific, Waltham, MA, USA). All reactions were conducted in duplicate and executed according to the thermal cycling conditions recommended by the manufacturer. Assay specificity was confirmed through melt-curve analysis, and amplification efficiency for each primer pair was estimated using standard curves, resulting in efficiencies ranging from 97% to 103%.

### 2.6. Evaluation of Reference Gene Expression Stability

NormFinder and RefFinder were selected, along with geNorm and BestKeeper, to evaluate the expression stability of candidate housekeeping genes (HKGs). NormFinder employs a model-based approach to estimate intra- and inter-group variation, yielding a stability value where lower scores indicate greater consistency [51]. GeNorm calculates the pairwise variation (M-value) between genes, ranks candidates, and estimates the optimal number required for reliable normalization [52]. BestKeeper is based on the raw Ct values of candidate reference genes, determining the standard deviation and coefficient of variation. A higher correlation with the BestKeeper Index indicates greater stability [53]. RefFinder integrates the results from NormFinder, geNorm, BestKeeper, and the comparative ΔCt method to provide a comprehensive stability ranking [54]. By combining multiple algorithms, this approach ensures a robust and reliable selection of reference genes under the experimental conditions of the study.

### 2.7. Statistical Analysis

Data analysis and visualization were performed using GraphPad Prism 10.5.0 (774) Software LLC (Boston, MA, USA). Descriptive statistics were used to calculate the mean and standard deviation (SD) of ΔCt values, while the standard error of the mean (SEM) was determined for raw Ct values. Data normality was assessed using the Kolmogorov–Smirnov test. Variables that did not meet the assumptions of normal distribution were analyzed using the non-parametric Kruskal–Wallis test, whereas normally distributed data were evaluated by one-way analysis of variance (ANOVA), followed by Tukey’s post hoc test for multiple comparisons. All graphical data are presented as mean ± SEM.

## 3. Results

### 3.1. Morphological Assessment of AML12 Cells Cultured in 2D and 3D Systems at Day 7

**Figure 1:**
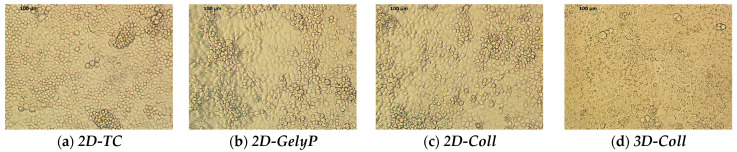
Representative morphology of AML12 cells cultured under 2D and 3D conditions at day 7 (20× magnification). Cells were maintained in four culture setups: (**a**) 2D standard tissue culture plate (2D-TC); (**b**) 2D thin-layer gelysate-peptone coating (2D-GelyP); (**c**) 2D thin-layer collagen type I coating (2D-Coll); (**d**) 3D collagen type I sandwich culture (3D-Coll). Distinct differences in cell morphology, spatial organization, and cell–cell interactions are observed depending on culture dimensionality and extracellular matrix context.

The images, demonstrating cell morphology and organization after 7 days of culture, are shown in Figure 1. AML12 cells cultured under 2D conditions, regardless of the adhesion matrix used, exhibited a relatively uniform distribution with occasional cluster formations, but lacked a distinct architectural organization characteristic of hepatic epithelial cells. Most of the cells displayed a more rounded morphology, likely reflecting limited cellular polarization.

In contrast, AML12 cells cultured under 3D conditions appeared markedly more homogeneous, exhibiting a well-defined polygonal morphology typical of hepatocytes. A higher degree of spatial organization was evident, with localized regions forming structures reminiscent of mini-lobules. Cell overlap was minimal or nearly absent.

### 3.2. Stability Evaluation of HKGs Expression Quantities Using Classical Algorithms—Normfinder and geNorm Excel-Based Tools

**Figure 2:**
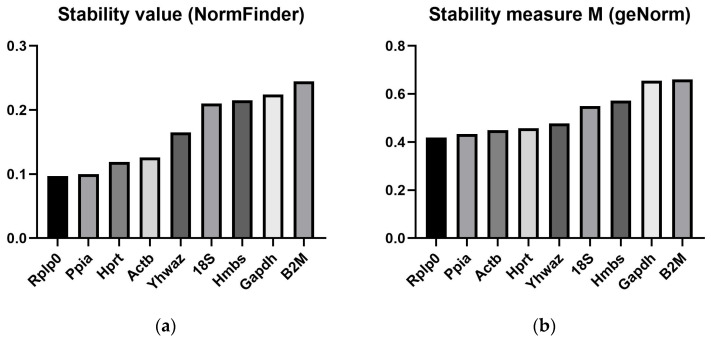
Stability ranking of candidate housekeeping genes in AML12 cells based on (**a**) NormFinder and (**b**) geNorm algorithms. The *y*-axis represents the stability value (NormFinder) or stability measure M (geNorm), where lower values indicate higher expression stability. Analysis was performed using efficiency-corrected RT-qPCR data, with amplification efficiencies ranging from 97% to 103%. Gene abbreviations are defined in Table 1.

The data presented in Figure 2a,b depict a highly similar stability profile of the evaluated HKGs. Both analyses—using the Excel-based NormFinder and geNorm tools—were performed with efficiency-corrected relative quantities. The RT-qPCR amplification efficiencies varied between 97% and 103%, so these differences did not substantially affect the obtained results. As shown in the figures, Rplp0, Ppia, Hprt, and Actb exhibited the lowest variability, while Ywhaz displayed an intermediate level of variation. In contrast, 18S, Hmbs, Gapdh, and B2M were identified as the most unstable genes.

### 3.3. Integrated Calculation of Candidate Reference Gene Expression Stability

**Figure 3:**
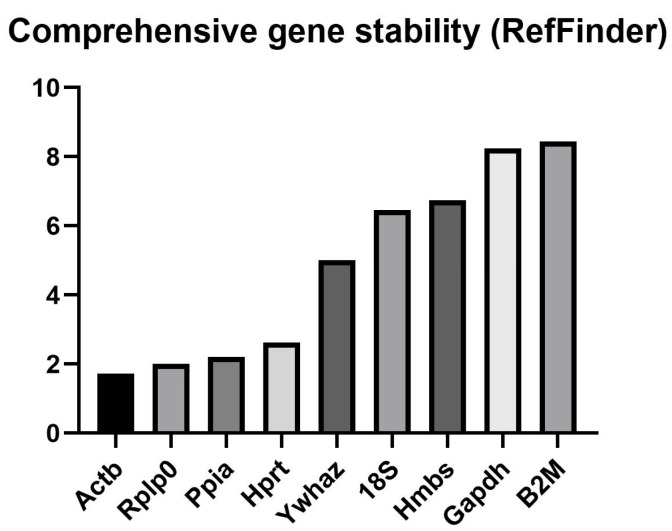
Comprehensive ranking of candidate reference genes in AML12 cells generated by RefFinder based on Ct values. The *y*-axis represents the overall ranking score derived from the integration of multiple algorithms (ΔCt method, BestKeeper, NormFinder, and geNorm). Lower ranking scores indicate higher expression stability, whereas higher values reflect reduced suitability as reference genes. Gene abbreviations are defined in Table 1.

The RefFinder software (https://www.ciidirsinaloa.com.mx/RefFinder-master/, accessed on 21 October 2024) tool enables the simultaneous evaluation of gene expression stability using the commonly applied methods: BestKeeper, NormFinder, geNorm, and the comparative ΔCt method described by Silver et al. [55]. However, RefFinder analyzes raw Ct values and assumes a default RT-qPCR amplification efficiency of 100%. Therefore, in the comparison of the obtained results method by method, slight discrepancies were observed for NormFinder and geNorm when referred to the original tool’s data. Nevertheless, the summarized RefFinder output also identified Actb, Rplp0, Ppia, and Hprt as the most stable reference genes, whereas 18S, Hmbs, Gapdh, and B2M exhibited the highest variability, with approximately three- to four-fold greater instability (Figure 3).

### 3.4. Estimation of Candidate Reference Gene Stability Using the ΔCt Method

Based on the analysis of the SD of ΔCt values presented in Table 2, where the expression of each investigated HKG was normalized against all others in accordance with the method described by Silver et al. [55], three distinct levels of expression stability could be identified. The most stable genes exhibited mean SD values ranging from 0.41 to 0.48 and included Actb, Rplp0, Ppia, Hprt, and Ywhaz. Intermediate stability was observed for 18S rRNA and Hmbs, with mean SD values of approximately 0.54. The highest variability was detected for Gapdh and B2M, with the mean SD of ΔCt values exceeding 0.6.

### 3.5. Statistical Calculation of Candidate Reference Genes and the Target Gene Albumin Using the BestKeeper Algorithm

All analyzed genes, evaluated using the BestKeeper tool, exhibited an SD below 1 (Table 3), fulfilling the original BestKeeper criterion for stable reference genes (Pfaffl et al., 2004 [53]). Most genes showed SD values below 0.5, which meets the established threshold for highly stable internal reference genes. The lowest variability was observed for Actb, Ppia, and Hprt (SD ≤ 0.22), followed by Rplp0 and Ywhaz (SD ≤ 0.28), confirming their high expression consistency across all experimental conditions. Hmbs, B2M, and 18S rRNA also demonstrated acceptable stability (SD < 0.5), despite higher CV values. Gapdh displayed the highest variability (SD = 0.55; CV = 3.04%), raising concerns about its suitability as a reference gene in the present experimental model.

Regression analysis against the BestKeeper index (BK), presented in Table 4, revealed that none of the investigated HKGs showed a strong correlation (r > 0.9) with the BK. Rplp0 exhibited the highest positive correlation (r = 0.83), supporting its high expression stability. Gapdh and 18S rRNA also showed relatively strong correlations (r ≥ 0.78), but for Gapdh, this finding contrasted with its higher variability observed in the SD-based analysis. Notably, several genes with low SD values (Actb, Ppia, Hprt) displayed weaker or negative correlations with the BK, while the target gene Albumin showed a significant negative correlation with BK.

### 3.6. Pearson Correlation Analysis Between Each Housekeeping Gene and Albumin

Pearson correlation analysis revealed a strong to moderate, statistically significant correlation between albumin and *Ywhaz* (r = −0.733, *p* < 0.001), *Rplp0* (r = −0.490, *p* < 0.015), и *Gapdh* (r = −0.427, *p* < 0.037) and a lack of statistically significant relation of Albumin with Actb, 18S, B2M, Hmbs, Hprt, or Ppia (*p* > 0.05) (Table 5).

### 3.7. Comparative Intergroup Analysis of Raw Ct Values for Each Candidate HKG

**Figure 4:**
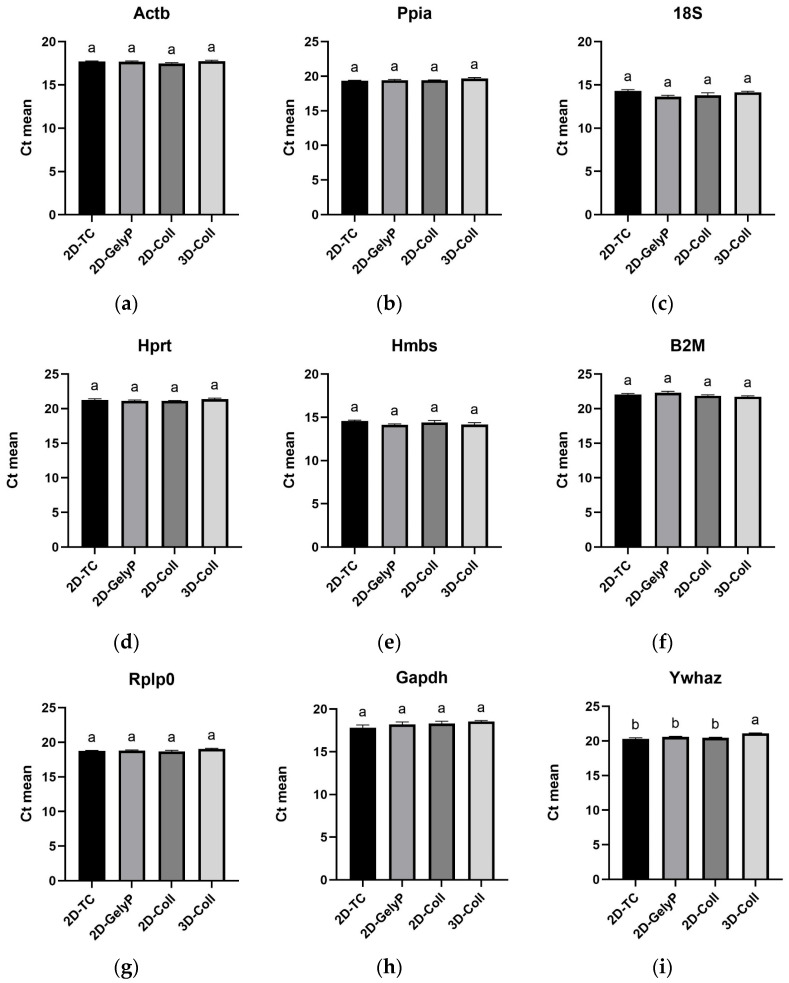
Mean Ct values of candidate housekeeping genes in AML12 cells cultured under different 2D and 3D conditions. Panels (**a**–**i**) correspond to (**a**) Actb, (**b**) Ppia, (**c**) 18S, (**d**) Hprt, (**e**) Hmbs, (**f**) B2M, (**g**) Rplp0, (**h**) Gapdh, and (**i**) Ywhaz. Cells were maintained in four culture setups: 2D standard tissue culture plate (2D-TC); 2D thin-layer gelysate-peptone coating (2D-GelyP); 2D thin-layer collagen type I coating (2D-Coll); 3D collagen type I sandwich culture (3D-Coll). Bars represent mean Ct values. Different letters above the bars indicate statistically significant differences between groups (*p* < 0.05).

Figure 4 shows a comparative intergroup analysis of raw Ct values for all tested candidate HKGs to identify statistically significant differences between experimental conditions. For most genes, no significant differences in Ct values were found among groups, indicating stable expression regardless of culture format. The only exception was Ywhaz, which was significantly downregulated under 3D conditions in contrast to all 2D models, suggesting that its expression is sensitive to cellular spatial organization.

### 3.8. Graphical Representation of Albumin ΔCt Values Normalized to Individual Housekeeping Genes in Relation to Raw Ct Values

**Figure 5:**
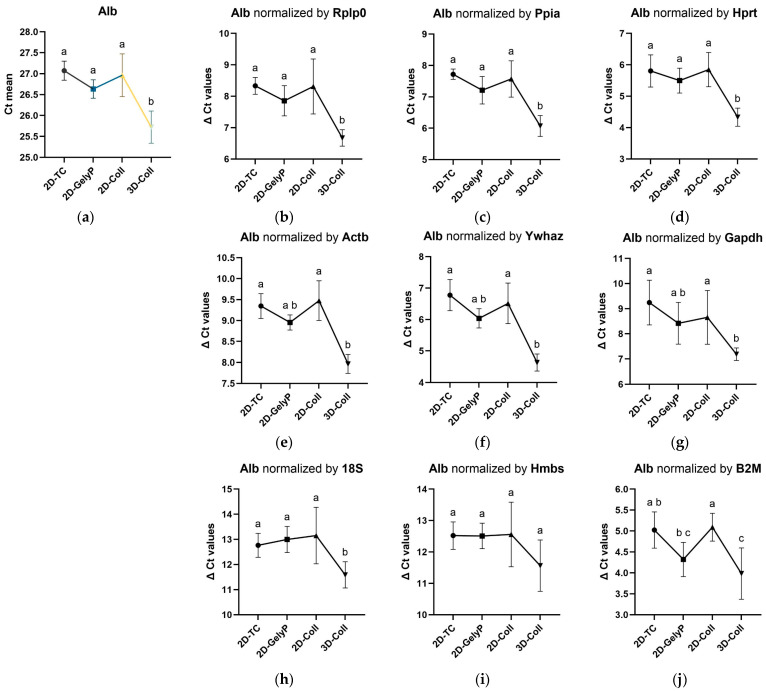
Albumin (Alb) levels in AML12 cells cultured under different 2D and 3D conditions, shown as raw Ct values and after normalization to individual housekeeping genes (HKGs). Panels (**a**–**j**) represent (**a**) raw Alb Ct mean values and Alb normalized to (**b**) Rplp0, (**c**) Ppia, (**d**) Hprt, (**e**) Actb, (**f**) Ywhaz, (**g**) Gapdh, (**h**) 18S, (**i**) Hmbs, and (**j**) B2M, presented as ΔCt values. Cells were maintained in four culture setups: 2D standard tissue culture plate (2D-TC); 2D thin-layer gelysate-peptone coating (2D-GelyP); 2D thin-layer collagen type I coating (2D-Coll); 3D collagen type I sandwich culture (3D-Coll). Different letters above the data points indicate statistically significant differences between groups (*p* < 0.05).

The graphical representation of the raw Ct values of Albumin and the corresponding ΔCt values, normalized to the individual HKGs, demonstrates a clear reduction in Albumin expression under 3D culture conditions compared to all 2D models (Figure 5). This pattern is preserved when normalized to most reference genes (Rplp0, Ppia, Hprt, Actb, Ywhaz, Gapdh), with statistically significant differences between 2D and 3D conditions remaining relatively consistent. In contrast, normalization to 18S, Hmbs, and B2M results in greater variability, changes, or partial loss of statistical significance, reflecting their lower stability as internal controls.

### 3.9. Final Validation of Preselected Stable Reference Genes and Normalization Outcomes

**Figure 6:**
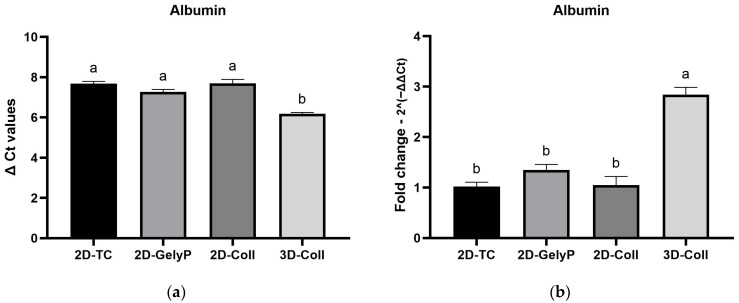
Albumin gene expression normalized to the geometric mean of the selected stable reference genes (Hprt, Ppia, and Actb): (**a**) Relative expression based on ΔCt values and (**b**) Fold change calculated using the ΔΔCt method. Statistically significant differences between groups are indicated by different letters (*p* < 0.05).

Based on the comprehensive stability analyses, Hprt, Ppia, and Actb were selected as the most stable reference genes and were subsequently used for the final BestKeeper analysis (Table 6 and Table 7) and normalization of Albumin expression (Figure 6). According to the BestKeeper calculations, all three genes exhibited exceptionally high expression stability, with SD values below 0.23 and CV below 1.10. In the regression analysis against the BK, these reference genes showed strong and highly significant correlations (r > 0.68, *p* < 0.001), while no interaction was observed between the BK and the target gene Albumin, which supports a correct and independent evaluation of the RT-qPCR data.

Normalization was performed using the geometric mean of the three selected reference genes. Relative expression levels were calculated using the 2^−ΔΔCt^ method, with the 2D-TC group used as the calibrator. Albumin expression demonstrated a consistent increase under 3D-Coll conditions compared to 2D cultures, whereas intermediate expression levels were observed in the 2D-GelyP and 2D-Coll groups. This expression pattern closely mirrors the trends observed in the raw Ct data, as illustrated in Figure 5.

## 4. Discussion

Several studies have demonstrated that the transition from 2D to 3D cell culturing substantially alters the stability of the commonly used housekeeping genes [56,57,58]. For example, Rplp0 and Gapdh were identified as the most stable reference genes under 2D conditions, while their stability often decreases under 3D conditions. In contrast, Ppia, Hprt and Ywhaz emerged as one of the most suitable candidates in 3D models, depending on the cell type and employed culture system [34,59,60,61]. Similar culture-dependent differences have also been observed in hepatocellular models such as Huh-7, identifying Ppia and Rpl13 as the most appropriate reference genes under 3D conditions [57]. However, systematic validation of housekeeping genes in 3D cultures of AML12 cells is currently lacking, and most methodological studies rely on a single reference gene, such as Rplp0, without assessing its stability across different culture conditions [62,63,64]. In this context, our results provide direct evidence that reference gene stability in AML12 cells is strongly influenced by the culture dimensionality, underscoring the need for condition-specific validation prior to RT-qPCR normalization [65].

In our previous studies, we demonstrated that combining classical algorithms for assessing the stability of reference genes with independent statistical methods leads to a significantly more reliable and reasoned selection of housekeeping genes for normalization [66]. The present study confirms this concept and clearly reveals that the simultaneous use of algorithmic and statistical criteria enables a more precise elimination of genes that would compromise the final RT-qPCR analysis. In the first stage, we applied the commonly used tools NormFinder, geNorm, RefFinder, and ΔCt analysis. All of them gave similar results, identifying Rplp0, Ppia, Hprt, and Actb as the most stable candidates for normalization, while 18S, Hmbs, Gapdh, and B2M consistently ranked as significantly less stable.

The increased variability observed in Gapdh and B2M expression is likely associated with local differences in cell density and cluster formation in 2D cultures. Irregular cell distribution and focal cell aggregation under these conditions most probably generate microenvironmental and metabolic heterogeneity, leading to altered regulation of specific reference genes [67,68]. B2M, as an integral component of the MHC class I complex, is particularly sensitive to changes in cellular state and microenvironment, whereas Gapdh, a key glycolytic enzyme, is well known to exhibit variable expression in response to fluctuations in oxygen availability, glucose supply, and overall metabolic activity [38,69]. On the other hand, the 3D collagen sandwich model promotes a more even spatial organization of cells, reducing variability driven by density. Consequently, the overall stability expression of these commonly used control genes was compromised under our experimental conditions.

In the present experiment, 18S rRNA and Hmbs showed significantly higher expression levels compared to the other investigated genes. In the case of 18S rRNA, this likely reflects its distinct biological nature, as it is a ribosomal RNA with a very high abundance, different transcriptional dynamics, and a significantly wider dynamic range compared to mRNA transcripts. These characteristics may lead to increased sensitivity to slight variations in the amount of input RNA and reverse transcription efficiency, as well as to limited comparability with mRNA targets. Such obstructions in using 18S rRNA as a reference gene have been widely described in the literature and are considered a significant drawback in the normalization of RT-qPCR data [70].

Herein, Ywhaz was ranked as a moderately stable gene, occupying an intermediate position in the overall stability assessment. Ywhaz is frequently described in the literature as a highly stable HKG [65,71]. Still, it was unexpectedly the only candidate that exhibited a statistically significant reduction in expression under 3D conditions, thereby disqualifying it as a stable internal control in this experimental setting. The observed decrease in Ywhaz expression in the 3D collagen sandwich model likely reflects a biologically driven change in cellular phenotype rather than technical instability of the reference gene. Ywhaz encodes 14-3-3ζ, an adaptor protein involved in cell cycle regulation, including the negative control of the G2–M transition through the cytoplasmic sequestration of cyclin-dependent kinases, resulting in the inhibition of their activity [72]. Under 3D collagen type I culture conditions, AML12 cells appear to regain features consistent with a more physiologically relevant polarization and reduced proliferative activity. These observations, based on microscopic examination, may reasonably suggest a decreased requirement for 14-3-3ζ-mediated regulation. In this context, reduced Ywhaz expression may be interpreted as an indicator of a shift toward a more functionally stable hepatocyte-like phenotype.

This finding highlights a key principle: even a gene with favorable stability rankings may be unsuitable as a reference gene if it displays biologically driven differences between experimental groups. This apparent discrepancy can be explained by the fact that stability algorithms primarily assess intra-group variability rather than biological invariance. When a gene exhibits low fluctuation within each group but undergoes a consistent, condition-dependent shift between experimental groups, it may be incorrectly classified as stable. Importantly, without classical intergroup statistical analysis, this variability would have remained undetected. Therefore, intergroup statistical testing should be considered an essential requirement for validating reference genes in RT-qPCR analyses.

The BestKeeper analysis also warns about a problem with the “stable panel” of genes. Often, authors base their selection solely on the statistical analysis from BestKeeper, without performing a regression analysis, which requires a target gene to be specified. If we had referred to the results based only on SD and CV, in our case, the following genes would again qualify as stable (SD < 0.5): Actb, Ppia, Hprt, Rplp0, and Ywhaz, in accordance with the previously evaluated algorithm’s data. However, the regression analysis revealed an important problem: all of the genes, without Actb, are statistically significantly correlated with the overall BestKeeper index. However, we found a moderate, statistically significant correlation between the BestKeeper Index and the target gene, indicating that normalization with these genes would lead to a systematic bias in the results. This is a key finding that is often overlooked in routine analyses. The subsequent Pearson correlation analysis performed in GraphPad Prism revealed a significant relationship between Rplp0, Gapdh, and Ywhaz and the target gene-Albumin, which automatically eliminated them as potential normalization factors. This result is particularly important because, although Rplp0 was ranked as the most stable gene by NormFinder, geNorm, and BestKeeper regression analysis, its correlation with the target gene suggests compromised biological stability under the specific experimental conditions, which could lead to misleading results. The inclusion of housekeeping genes that correlate with the target gene, as well as highly variable candidates, can distort the BestKeeper index. Under such conditions, correlation undermines the fundamental logic of normalization and fails to ensure proper panel stability. Therefore, potentially confounding reference genes should be excluded before performing regression analysis in BestKeeper.

Based on the comprehensive stability assessment and the combined stepwise elimination strategy, Hprt, Ppia, and Actb were identified as the most reliable reference genes in the present experimental model, demonstrating extremely low intra-group variability, absence of significant intergroup differences, and strong agreement with the BestKeeper index. The lack of interaction between the BestKeeper index and the target gene Albumin further confirms that normalization using these reference genes enables an accurate and independent evaluation of RT-qPCR data. Application of the geometric mean of the three reference genes in ΔCt and ΔΔCt analyses revealed a consistent increase in Albumin expression in the 3D collagen model compared with 2D cultures, while intermediate expression levels were observed under 2D_GelP and 2D_Coll conditions. This expression pattern closely mirrors the trends noticeable in the raw Ct values, further validating the robustness of the analytical approach applied.

## 5. Conclusions

The present study demonstrates that the assessment of gene expression in 2D and 3D AML12 in vitro models, requires a context-dependent and critical selection of reference genes, as changes in cellular organization and microenvironment can affect even widely used housekeeping genes’ stability. Based on an integrated stability assessment and exclusion-based analysis, Hprt and Ppia are recommended as the most reliable reference genes for RT-qPCR normalization in AML12 cells under both 2D and collagen-based 3D culture conditions. Actb representing an acceptable additional candidate. Ywhaz and Rplp0 are not recommended due to their correlation with Albumin expression and culture-dependent regulation. B2M, Gapdh, 18S, and Hmbs are not acceptable due to their increased variability. Overall, this study emphasizes the importance of systematic, condition-specific validation of reference genes and offers a robust framework for reliable RT-qPCR normalization when comparing 2D and 3D in vitro culture systems.

## Figures and Tables

**Table 1:** Nucleotide sequences of primer pairs for housekeeping and target genes employed in RT-qPCR.

Abbreviation	Full Name	Forward Primer	Reverse Primer	Product Size (bp)
B2M NM_009735.3	Beta-2 microglobulin	TGTATGCTATCCAGAAAACCCCT	TTTCAATGTGAGGCGGGTGG	117
Ppia NM_008907.2	Peptidylprolyl isomerase A	GAACATTGTGGAAGCCATGGAG	AGATGGGGTAGGGACGCTC	163
Gapdh NM_001289726.2	Glyceraldehyde-3-phosphate dehydrogenase	TCAGGAGAGTGTTTCCTCGTC	TCGTGGTTCACACCCATCAC	439
Ywhaz NM_001356569.1	Tyrosine 3-monooxygenase/tryptophan 5-monooxygenase activation protein, zeta polypeptide	AGACGGAAGGTGCTGAGAAA	TTGTCATCACCAGCAGCAAC	211
Hmbs NM_001110251.1	Hydroxymethylbilane synthase	CCTGAAGGATGTGCCTACCA	CCACTCGAATCACCCTCATCT	175
Rplp0 NM_007475.5	Ribosomal protein, large, P0	TTATAACCCTGAAGTGCTCGAC	CGCTTGTACCCATTGATGATG	147
Hprt NM_013556.2	Hypoxanthine guanine phosphoribosyl transferase	ACAGGCCAGACTTTGTTGGA	ACTTGCGCTCATCTTAGGCT	150
Actb NM_007393.5	β-actin	CCTCTATGCCAACACAGTGC	GTACTCCTGCTTGCTGATCC	211
18S NR_046271.1	18S ribosomal RNA	ATGCGGCGGCGTTATTCC	GCTATCAATCTGTCAATCCTGT	204
Alb NM_009654.4	Mus musculus, albumin	AGCCTGCCACCATTTGAAAG	TTCACACCATCAAGCTTCGG	227

**Table 2:** ΔCt-derived evaluation of expression consistency among candidate reference genes.

Gene Names	Mean ΔCt	SD	Mean SD*	Gene Names	Mean ΔCt	SD	Mean SD*
Actb/Rplp0	1.14	0.35		18S/Hmbs	0.45	0.30	
Actb/18S	3.69	0.55		18S/Hprt	7.258	0.53	
Actb/B2M	4.33	0.41		18S/Ppia	5.48	0.55	
Actb/Gapdh	0.77	0.45		18S/Ywhaz	6.64	0.54	0.53 ^a b^
Actb/Hmbs	3.35	0.57		Ppia/Actb	1.79	0.31	
Actb/Hprt	3.57	0.28		Ppia/Rplp0	0.65	0.27	
Actb/Ppia	1.79	0.31		Ppia/18S	5.48	0.55	
Actb/Ywhaz	2.94	0.38	0.41 ^b^	Ppia/B2M	2.54	0.57	
Hprt/Actb	3.57	0.28		Ppia/Gapdh	1.23	0.59	
Hprt/Rplp0	2.42	0.33		Ppia/Hmbs	5.14	0.55	
Hprt/18S	7.26	0.50		Ppia/Hprt	1.77	0.34	
Hprt/B2M	0.79	0.51		Ppia/Ywhaz	1.15	0.33	0.44 ^a b^
Hprt/Gapdh	3.01	0.65		Gapdh/Actb	0.77	0.45	
Hprt/Hmbs	6.92	0.55		Gapdh/Rplp0	0.59	0.52	
Hprt/Ppia	1.77	0.34		Gapdh/18S	4.25	0.59	
Hprt/Ywhaz	0.62	0.45	0.45 ^a b^	Gapdh/B2M	3.77	0.96	
Rplp0/Actb	1.14	0.35		Gapdh/Hmbs	3.91	0.62	
Rplp0/18S	4.83	0.43		Gapdh/Hprt	3.01	0.65	
Rplp0/B2M	3.19	0.60		Gapdh/Ppia	1.24	0.60	
Rplp0/Gapdh	0.59	0.52		Gapdh/Ywhaz	2.39	0.50	0.61 ^a b^
Rplp0/Hmbs	4.50	0.49		Ywhaz/Actb	2.94	0.39	
Rplp0/Hprt	2.42	0.33		Ywhaz/Rplp0	1.80	0.34	
Rplp0/Ppia	0.65	0.27		Ywhaz/18S	6.64	0.54	
Rplp0/Ywhaz	1.80	0.34	0.42 ^b^	Ywhaz/B2M	1.39	0.65	
Hmbs/Actb	3.36	0.57		Ywhaz/Gapdh	2.39	0.49	
Hmbs/Rplp0	4.50	0.49		Ywhaz/Hmbs	6.30	0.61	
Hmbs/18S	0.45	0.30		Ywhaz/Hprt	0.62	0.45	
Hmbs/B2M	7.69	0.70		Ywhaz/Ppia	1.15	0.33	0.48 ^a b^
Hmbs/Gapdh	3.91	0.62		B2M/Actb	4.33	0.41	
Hmbs/Hprt	6.92	0.55		B2M/Rplp0	3.19	0.60	
Hmbs/Ppia	5.15	0.55		B2M/18S	8.02	0.79	
Hmbs/Ywhaz	6.30	0.61	0.55 ^a b^	B2M/Gapdh	3.77	0.96	
18S/Actb	3.69	0.55		B2M/Hmbs	7.69	0.70	
18S/Rplp0	4.84	0.43		B2M/Hprt	0.79	0.51	
18S/B2M	8.02	0.79		B2M/Ppia	2.54	0.57	
18S/Gapdh	4.25	0.59		B2M/Ywhaz	1.39	0.66	0.65 ^a^

SD represents the standard deviation of ΔCt values for each gene pair. SD* represents the mean standard deviation of pairwise ΔCt values calculated across all combinations involving the respective reference gene and serves as an indicator of expression stability; Different letters indicate statistically significant differences among SD* (*p* < 0.05).

**Table 3:** Statistical assessment of candidate reference genes and the target gene Albumin based on the Bestkeeper algorithm.

	*Actb*	*Ppia*	*Hprt*	*Rplp0*	*Ywhaz*	*Hmbs*	*B2M*	*18S*	*Gapdh*	*Albumin*
n	24	24	24	24	24	24	24	24	24	24
geo Mean [CP]	17.66	19.45	21.23	18.80	20.60	14.30	21.99	13.96	18.21	26.59
ar Mean [CP]	17.66	19.46	21.23	18.81	20.61	14.31	21.99	13.97	18.22	26.60
min [CP]	17.23	19.08	20.86	18.12	19.61	13.15	21.33	13.10	16.77	25.07
max [CP]	17.99	20.10	21.87	19.43	21.26	15.11	22.85	14.83	18.98	27.67
std dev [± CP]	0.18	0.21	0.22	0.28	0.28	0.34	0.35	0.41	0.55	0.50
CV [% CP]	1.03	1.10	1.04	1.47	1.35	2.35	1.61	2.96	3.04	1.89

std dev [±CP] represents the standard deviation of Cp (Ct) values across all samples for each gene. CV [% CP] represents the coefficient of variation calculated from Cp values. Power [x-fold] indicates the expression range of each gene, calculated as the fold difference between maximum and minimum Cp values.

**Table 4:** Linear regression analysis of candidate reference genes and the target gene Albumin relative to the BestKeeper index.

	*Rplp0* vs. *BK*	*Gapdh* vs. *BK*	*18S* vs. *BK*	*Ywhaz* vs. *BK*	*Hmbs* vs. *BK*	*Ppia* vs. *BK*	*Hprt* vs. *BK*	*B2M* vs. *BK*	*Actb* vs. *BK*	*Albumin* vs. *BK*
coeff. of corr. [r]	0.83	0.794	0.779	0.596	0.548	0.463	0.422	−0.411	0.193	−0.466
coeff. of det. [r^2]	0.689	0.63	0.607	0.355	0.3	0.214	0.178	0.169	0.037	0.217
intercept [CP]	−4.53	−25.194	−19.437	1.694	−6.66	8.763	10.623	36.573	14.082	51.731
slope [CP]	1.278	2.377	1.829	1.036	1.148	0.585	0.581	−0.798	0.196	−1.376
SE [CP]	±0.189	±0.401	±0.324	±0.307	±0.386	±0.247	±0.275	±0.39	±0.22	±0.575
*p*-value	0.001	0.001	0.001	0.002	0.006	0.023	0.04	0.046	0.368	0.022
Power [x-fold]	2.46	5.32	3.52	2.05	2.22	1.51	1.5	0.57	1.15	0.39

**Table 5:** Pearson correlation coefficients and *p*-values between individual HKG and Albumin.

*Albumin* vs.	*Actb*	*Rplp0*	*18S*	*B2M*	*Gapdh*	*Hmbs*	*Hprt*	*Ppia*	*Ywhaz*
n	24	24	24	24	24	24	24	24	24
Pearson (r) correlation	0.004	−0.490	−0.275	0.314	−0.427	−0.048	−0.199	−0.294	−0.733
*p*-value	0.986	0.015	0.194	0.135	0.037	0.825	0.352	0.164	<0.001

**Table 6:** BestKeeper statistical parameters of the preselected stable reference genes.

	*Actb*	*Ppia*	*Hprt*	*Albumin*
n	24	24	24	24
geo Mean [CP]	17.66	19.45	21.23	26.59
ar Mean [CP]	17.66	19.46	21.23	26.60
min [CP]	17.23	19.08	20.86	25.07
max [CP]	17.99	20.10	21.87	27.67
std dev [± CP]	0.18	0.21	0.22	0.50
CV [% CP]	1.03	1.10	1.04	1.89

**Table 7:** BestKeeper linear regression analysis of the preselected stable reference genes.

	*Hprt* vs. *BK*	*Ppia* vs. *BK*	*Actb* vs. *BK*	*Albumin* vs. *BK*
n	24	24	24	24
coeff. of corr. [r]	0.794	0.726	0.686	−0.230
coeff. of det. [r^2]	0.630	0.527	0.471	0.053
intercept [CP]	−2.589	1.571	0.589	41.382
slope [CP]	1.228	0.830	0.973	−0.762
SE [CP]	±0.184	±0.154	±0.202	±0.633
*p*-value	0.001	0.001	0.001	0.279
Power [x-fold]	2.343	1.792	1.982	0.592

## Data Availability

The original contributions presented in this study are included in the article. Further inquiries can be directed to the corresponding author.

## Abbreviations

The following abbreviations are used in this manuscript:

18S	18S ribosomal RNA
2D	two-dimensional
2D-GelyP	Cells cultured on a surface coated with 0.1% gelatin-peptone surface
2D-Coll	Cells cultured on a coated surface with a thin layer of type I collagen surface
2D-TC	Cells cultured on standard tissue culture-treated plastic surfaces
3D	three-dimensional
3D-Coll	Three-dimensional sandwich method of cultivation in collagen type I
3Rs	Reduction, Refinement, and Replacement
Alb	Albumin
AML12	Mouse hepatocytes alpha mouse liver 12
Actb	β-actin
B2M	Beta-2 microglobulin
BK	BestKeeper index
cDNA	Complementary deoxyribonucleic acid
Ct	Cycle threshold
ΔCt	delta Ct
ΔΔCt	delta delta Ct
CV	coefficient of variation
DMEM	Dulbecco’s Modified Eagle’s Medium
Gapdh	Glyceraldehyde-3-phosphate dehydrogenase
HKG	Housekeeping genes
Hmbs	Hydroxymethylbilane synthase
Hprt	Hypoxanthine guanine phosphoribosyl transferase
ITS	Insulin–transferrin–selenium
MHC I	Major histocompatibility complex class I
PBS	Phosphate-buffered saline
Ppia	Peptidylprolyl isomerase A
RNA	Ribonucleic acid
Rplp0	Ribosomal protein, large, P0
RT-qPCR	Reverse transcription quantitative polymerase chain reaction
SD	standard deviation
SEM	Standard error of the mean
TBP	TATA-box binding protein
Ywhaz	Tyrosine 3-monooxygenase/tryptophan 5-monooxygenase activation protein, zeta polypeptide
